# Bacterial Exchange in Household Washing Machines

**DOI:** 10.3389/fmicb.2015.01381

**Published:** 2015-12-08

**Authors:** Chris Callewaert, Sam Van Nevel, Frederiek-Maarten Kerckhof, Michael S. Granitsiotis, Nico Boon

**Affiliations:** ^1^Laboratory of Microbial Ecology and Technology, Department of Biochemical and Microbial Technology, Faculty of Bioscience Engineering, Ghent UniversityGhent, Belgium; ^2^Research Unit Environmental Genomics, Department of Environmental Science, Helmholtz Zentrum MünchenNeuherberg, Germany

**Keywords:** skin, fabrics, microbiome, domestic, washing machine

## Abstract

Household washing machines (WMs) launder soiled clothes and textiles, but do not sterilize them. We investigated the microbial exchange occurring in five household WMs. Samples from a new cotton T-shirt were laundered together with a normal laundry load. Analyses were performed on the influent water and the ingoing cotton samples, as well as the greywater and the washed cotton samples. The number of living bacteria was generally not lower in the WM eﬄuent water as compared to the influent water. The laundering process caused a microbial exchange of influent water bacteria, skin-, and clothes-related bacteria and biofilm-related bacteria in the WM. A variety of biofilm-producing bacteria were enriched in the eﬄuent after laundering, although their presence in the cotton sample was low. Nearly all bacterial genera detected on the initial cotton sample were still present in the washed cotton samples. A selection for typical skin- and clothes-related microbial species occurred in the cotton samples after laundering. Accordingly, malodour-causing microbial species might be further distributed to other clothes. The bacteria on the ingoing textiles contributed for a large part to the microbiome found in the textiles after laundering.

## Introduction

Bacteria enter the washing machine (WM) via worn clothing, household linen and influent water, while the laundry process is expected to deliver both visually and hygienically clean laundry (O’Toole et al., 2009; Teufel et al., 2010). In the past decades, laundry processes and detergents were substantially adjusted for environmental and economic reasons. The introduction of enzymes and the concomitant lower washing temperature, a decreased water consumption and the use of liquid detergents without disinfecting bleaching agents are some of the main adaptations in the laundry processes (Terpstra, 1998; Munk et al., 2001; Laitala and Jensen, 2010). These adjustments have impacted the hygienic quality of the laundry process.

In Europe, colored laundry is most often washed at temperatures between 30 to 40°C (Arild et al., 2003), offering good circumstances for bacteria to survive, or even, to grow. In China, South-Korea, Japan, and the USA, cold water is the most preferred water type (Pakula and Stamminger, 2010). Only an estimated 5% of the household laundering in the USA is done at 60°C or higher, an advised temperature for effectively killing possibly pathogenic bacteria (Munk et al., 2001; Bloomfield et al., 2007; Gerba and Kennedy, 2007). Any lower washing temperature offers survival conditions for bacteria and induces cross-contamination in the laundry. *Staphylococcus aureus* and *S. epidermidis*, for example, have been shown to survive laundry programs at 50°C (Munk et al., 2001).

A lower washing temperature of 40°C is only sufficient for disinfection when bleaches are used in the detergents (Bloomfield et al., 2007). These bleaches are mainly chlorine and peroxide based, with sodium hypochlorite being most used. They are still commonly added in hospital laundering, contrary to household laundering (Patel et al., 2006). Normal household detergents are developed primarily for removing dirt and stains, not for disinfection. At lower washing temperatures, the bleach activity strongly decreases and becomes insufficient (Ainsworth and Davis, 1989; Davis and Ainsworth, 1989). A thorough drying of the laundry can effectively decrease the bacterial load, although a slow drying process results in a considerable amount of bacterial growth, giving rise to malodour formation in the laundry (Munk et al., 2001).

The surviving bacteria build up biofilms in the WM and have higher resistance toward the used detergents. Modern machines often contain numerous plastic parts, which is ideal for adhesion and development of biofilms (Sheane, 2000). WM biofilms are shown to harbor many possible human pathogens like *Pseudomonas aeruginosa* and *Klebsiella pneumoniae*, sometimes even considerably more than toilets. Next to the pathogen risks, they induce malodour in WMs and freshly washed clothes (Munk et al., 2001; Gattlen et al., 2010). In order to counteract the biofilm build-up, most WM producers advice a maintenance wash, a monthly wash at high temperatures, preferably involving a bleaching agent.

**FIGURE 1:**
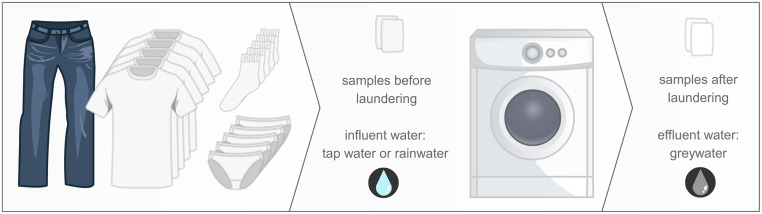
**Study design: one pair of jeans, five T-shirts, five pairs of socks, and five pieces of underwear, worn by the family members, were laundered using a delicate washing program, at 30°C, with a detergent without bleaching agents.**Samples were taken from the influent and eﬄuent water, and the unused cotton samples before and after laundering.

Despite the existing knowledge, the understanding of bacterial exchange in WMs remains inadequate. Next-generation sequencing is the new standard to characterize bacterial communities and offers an in-depth analysis of the microbiome (Turnbaugh et al., 2007). This study focused on gaining knowledge on the microbiome before and after a WM cycle and the possible cross-contamination within a WM. We characterized the bacterial communities of the influent and eﬄuent water of the WMs from five different families after running a similar washing program and load. Additionally, we included a newly bought cotton T-shirt and examined the microbial community of the fabric both before and after the laundering programs (**Figure 1**).

## Materials and Methods

### Experimental Design

The microbiome of five different household washing machines (WM 1–5) were studied (**Figure 1**). This study focussed on the bacterial flows throughout the WMs (no yeasts or fungi). To allow meaningful comparisons, a similar worn laundry load was washed on the same day. Samples were taken from the influent water (tap water or rainwater), the eﬄuent water after washing (greywater) and a cotton sample from a never worn T-shirt, before and after laundered in the WM. Absolute quantification (actual cell count) of bacterial cells was performed using the ‘old’ and ‘new’ standard technique: agar plating and flow cytometry. Relative quantification (ratio of specified sequencing read counts to total sequencing read counts) and identification was performed using the ‘old’ and ‘new’ standard technique: Denaturing Gradient Gel Electrophoresis (DGGE) and amplicon pyrosequencing. Descriptive α- and β-diversity analyses were performed on the results.

### Washing and Sampling

Washing machines of five different Belgian households located in East and West Flanders were studied (**Supplementary Table S1**). All members of these households were in good health and none of them had taken antibiotics for at least 1 month. The WMs were operated at a similar washing program: 30°C, delicate washing program, no pre-wash, centrifuging at 500 rpm, 45 g Le Chat powder detergent (Henkel, Germany), without fabric softener. The washing powder did not contain bleaching agents. A representative filling of the WM was obtained by laundering a load made up of colored textiles which have been worn by the household members: one pair of jeans, five pairs of socks, five pieces of underwear, and five T-shirts. The cross-contamination in the WM was studied by adding a 25 cm^2^ piece of a newly bought cotton T-shirt to these clothes. The washing was performed on the same day in Spring. After the completion of the complete washing program and during the last centrifugation, the first liter of greywater eﬄuent was discarded after which a sample was collected. The initial influent water used in the WM was also sampled. Tap water was used in two of the five WMs (WM 1–2), while the other three machines used rainwater (WM 3–5). The rainwater tanks and piping were localized underground; tap water originated from the Belgian public water supply, as well with underground piping. The cotton sample was analyzed before and after washing in an aseptic manner. After washing, the cotton sample was placed into a sealed plastic bag and stored at 4°C prior to analysis. Cotton was chosen as a representative clothing textile.

### Plating

A sequential series of 1:10 dilutions of the liquid samples was made in sterile saline solution (8.5 g/L NaCl) and plated by pour plating on Nutrient Agar (NA), Mc Conkey Agar (MCA), and Mannitol Salt Agar (MSA) to estimate total plate count (TPC), total Gram-negative bacteria, and total *Staphylococcus* and *Micrococcus* count, respectively. Incubation of all plates was performed for 72 h at 28°C under aerobic conditions.

### Flow Cytometry

For the counting of intact bacterial cells, two fluorescent dyes, SYBR^®^ Green I (SG) and propidium iodide (PI; Invitrogen, Belgium) were used for staining (Wang et al., 2010). When necessary, samples were diluted in 0.22 μm filtered bottled mineral water prior to staining. The staining solution was prepared as followed: PI (20 mM in dimethyl sulfoxide, DMSO) was diluted 50 times and SG (10,000 times concentrate in DMSO) was diluted 100 times in sterile DMSO. All samples were stained with 10 μl ml^-1^ staining solution and 10 μl ml^-1^ EDTA (pH 8, 500 mM) for outer membrane permeabilization. 10 μl ml^-1^ CytoCount counting beads (Dako, Belgium) were added as internal standard. Prior to flow cytometry analysis, the stained samples were incubated for 5 min in the dark at 37°C. Flow cytometry was performed using a Cyan^TM^ ADP LX flow cytometer as described by before (Boon et al., 2006). Only intact bacterial cells were counted. Dust particles and non-bacterial cells were excluded using the appropriate software.

### DNA Extraction

Liquid samples were brought into a 50 ml sterile reaction tube. 50 ml TNE buffer (10 mM Tris-HCl pH 8.0, 10 mM NaCl, 10 mM EDTA) was added to the sealed plastic bag with the cotton sample and vortexed for 10 min (Callewaert et al., 2014). The buffer was transferred into a 50 ml sterile reaction tube. DNA was extracted using the MoBio UltraClean Water DNA Isolation Kit (MO BIO Laboratories, Canada), according to manufacturer’s protocol. DNA samples were stored at –20°C until further processing. The DNA samples were used for DGGE and amplicon pyrosequencing.

### PCR and DGGE Analysis

The 16S rRNA gene region was amplified by PCR using 338F and 518R primers targeting the V3 region (Muyzer et al., 1993; Ovreas et al., 1997). A GC clamp of 40 bp (Muyzer et al., 1993; Ovreas et al., 1997) was added to the forward primer. The PCR program consisted of 10 min 95°C; 35 cycles of 1 min 94°C, 1 min of 53°C, 2 min of 72°C; and a final elongation for 10 min at 72°C. Amplification products were analyzed by electrophoresis in 1.5% (wt/vol) agarose gels stained with ethidium bromide. DGGE based on the protocol of Muyzer et al. (1993) was performed using the INGENYphorU System (Ingeny International BV, The Netherlands). PCR fragments were loaded onto 8% (w/v) polyacrylamide gels in 1 × TAE buffer (20 mM Tris, 10 mM acetate, 0.5 mM EDTA pH 7.4). To process and compare the different gels, a homemade marker of different PCR fragments was loaded on each gel (Boon et al., 2002). The polyacrylamide gels were made with denaturing gradients ranging from 40 to 60% (where 100% denaturant contains 7 M urea and 40% formamide). The electrophoresis was run for 16 h at 60°C and 120 V. Staining and analysis of the gels was performed as described previously (Boon et al., 2000). The normalization and analysis of DGGE gel patterns was done with the BioNumerics software 5.10 (Applied Maths, Sint-Martens-Latem, Belgium).

### PCR and 454 Pyrosequencing

Amplicon pyrosequencing was performed on a 454 XL+ Titanium system (Roche, Penzberg, Germany) as described before (Pilloni et al., 2012). Barcoded amplicons for multiplexing were prepared using the Ba27F and Ba519R primers, amplifying for the V1, V2, and V3 region of the 16S rRNA gene, extended with the respective A or B adapters, key sequence and multiplex identifiers (MID) as recommended by the manufacturer. Pyrotag PCR was performed in a Mastercycler ep gradient (Eppendorf, Hamburg, Germany) with the following cycling conditions: initial denaturation (94°C, 5 min), followed by 28 cycles of denaturation, annealing, and elongation (94°C – 30 s, 52°C – 30 s and 70°C – 60 s), followed by a final elongation (5 min – 70°C). For each sample the PCR reaction was performed in triplicates, in a final volume of 50 μl containing 1 × PCR buffer, 1.5 mM MgCl_2_, 0.1 mM dNTPs, 1.25 U recombinant Taq polymerase (Fermentas, St. Leon-Rot, Germany), 0.2 μg ml^-1^ bovine serum albumin (Roche), 0.3 mM of each MID-primer (Biomers, Ulm, Germany) and approximately 50 ng of template DNA. The triplicate amplicons were pooled together and purified using PCRExtract Mini kit (5 PRIME, Hilden, Germany) following the manufacturer instructions. Libraries were quantified by the Quant-iT PicoGreen dsDNA quantification kit (Invitrogen, Paisley, UK), diluted accordingly and pooled in an equimolar ratio of 10^9^ molecules ml^-1^. Emulsion PCR, emulsion breaking and sequencing were performed by applying the GS FLX Titanium chemistry following supplier protocols. Pyrosequencing was performed in a Picotiter Plate, in a pool with other samples, with 26 samples per quarter of a plate. The influent water, greywater and cotton samples of WM1, WM3, and WM4 as well as the unwashed cotton samples and the influent water of WM2 and WM5 were analyzed by means of amplicon pyrosequencing. The other samples did not succeed for PCR and pyrosequencing.

### 16S rRNA Gene Sequence Analysis

Quality filtering of the pyrosequencing reads was performed using the automatic amplicon pipeline of the GS Run Processor (Roche), with a modification of the valley filter (vfScanAll- Flows false instead of TiOnly) to extract sequences. The raw reads were further quality trimmed using the TRIM function of GreenGenes (DeSantis et al., 2006) with default settings. Flows shorter than 250 bp after trimming and with incorrect primers sequences were excluded from further analysis. Sequence classification was done for combined forward and reverse reads for each library using the RDP classifier (Wang et al., 2007). Prior of classification, chimeric sequences were removed using Uchime (Edgar et al., 2011) in an in-house Mothur and R/Sweave pipeline (Schloss et al., 2009). The non-chimeric sequences were classified using RDP’s command line MultiClassifier (version 2.7) tool (Cole et al., 2014) with confidence threshold set to 80%. Contigs of dominating amplicons were assembled with SEQMAN II software (DNAStar, Madison, WI, USA), using forward- and reverse-reads, as described before (Pilloni et al., 2011). Thresholds of read assembly into one contig were set to at least 98% sequence similarity for a minimum overlap of 50 bp. Contigs within one library with at least one forward and one reverse read were excluded from further analysis. Additional chimeric check was performed with Uchime as described. A total of 35152 sequence reads were obtained. The abundances per sample were calculated relative to the total sequence read per sample.

### Statistical Analysis

Statistical analysis was performed in SPSS (IBM Inc., USA) and significant cut-off values were set at the 95% confidence level (*p* < 0.05). Descriptive α- and β-diversity statistics were calculated using Mothur (Schloss et al., 2009) and visualized with R^1^. The α-diversity was calculated to characterize the diversity of one individual sample. The Chao 1 richness estimator and the Shannon diversity index were calculated using the RDP pyrosequencing pipeline (Cole et al., 2014). To assess the completeness of sampling also a rarefaction analysis was performed. The richness and evenness of the DGGE samples was estimated by means of the total band count and the Gini coefficient. The Gini coefficient was calculated based on the Pareto–Lorenz curve and was constructed based on the DGGE profiles (Marzorati et al., 2008). The β-diversity analysis was calculated to study the difference between the different microbial communities. Clustering of the DGGE samples was performed based on Pearson correlation and unweighted pair group with mathematical averages dendrogram method.

### Accession Codes

The sequences have been deposited in the NCBI Sequence Read Archive^2^ under study accession number SRR1619235.

## Results

### Bacterial Cell Counts

**FIGURE 2:**
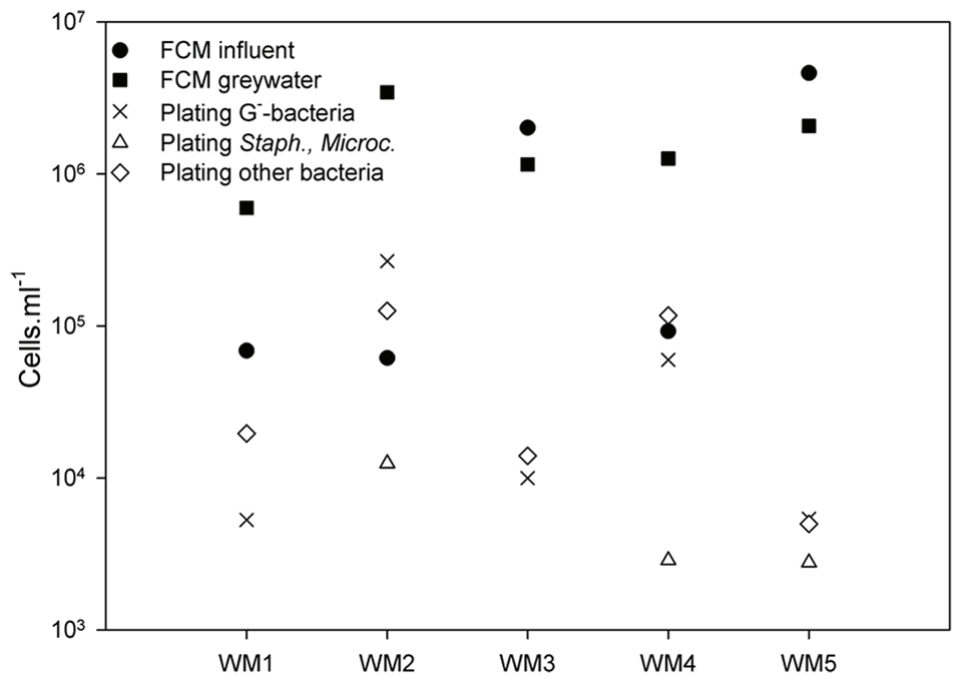
**Bacterial cell counts based on plating on NA (total plate count), MCA (Gram-negative bacteria), and MSA (staphylococci and *Micrococcaceae*) in the greywater samples; and intact bacterial cell counts based on flow cytometry (FCM) of the influent and greywater samples of the different washing machines (WM).**‘Other bacteria’ refer to all bacteria detected on NA, not detected on MCA and MSA.

Absolute bacterial abundances in the greywater were studied using TPC and selective plating, while the intact cells in both influent and greywater eﬄuent were counted by flow cytometry. Plating revealed a TPC between 1.32 × 10^4^ and 4.06 × 10^5^ culturable cells ml^-1^ (**Figure 2**). The total bacterial counts were the highest (10^5^ cells ml^-1^) for WM2 and WM4. The bacterial counts were a magnitude lower (10^4^ cells ml^-1^) for WM1, WM3, and WM5. The Gram-negative bacteria (Mc Conkey agar) accounted for 11 to 65% of the TPC-bacteria in the greywater. WM1 and WM3 contained no staphylococci or *Micrococcaceae* (measured by MSA agar), WM2 and WM4 contained 8 and 17%, respectively, while WM5 contained 60% staphylococci and *Micrococcaceae*. Using flow cytometry, we detected 7 to 157 times more intact cells in the greywater samples compared to plating (**Figure 2**). The two tap water samples had similar cell counts; 6.9 × 10^4^ and 6.1 × 10^4^ cells ml^-1^. The rainwater showed variable cell counts of 9.2 × 10^4^ up to 4.6 × 10^6^ cells ml^-1^. A higher cell number count was retrieved after washing, with 6.0 × 10^5^ to 3.4 × 10^6^ cells ml^-1^ present in the five greywater samples.

### Bacterial Community Analysis According to 454 Pyrosequencing

**FIGURE 3:**
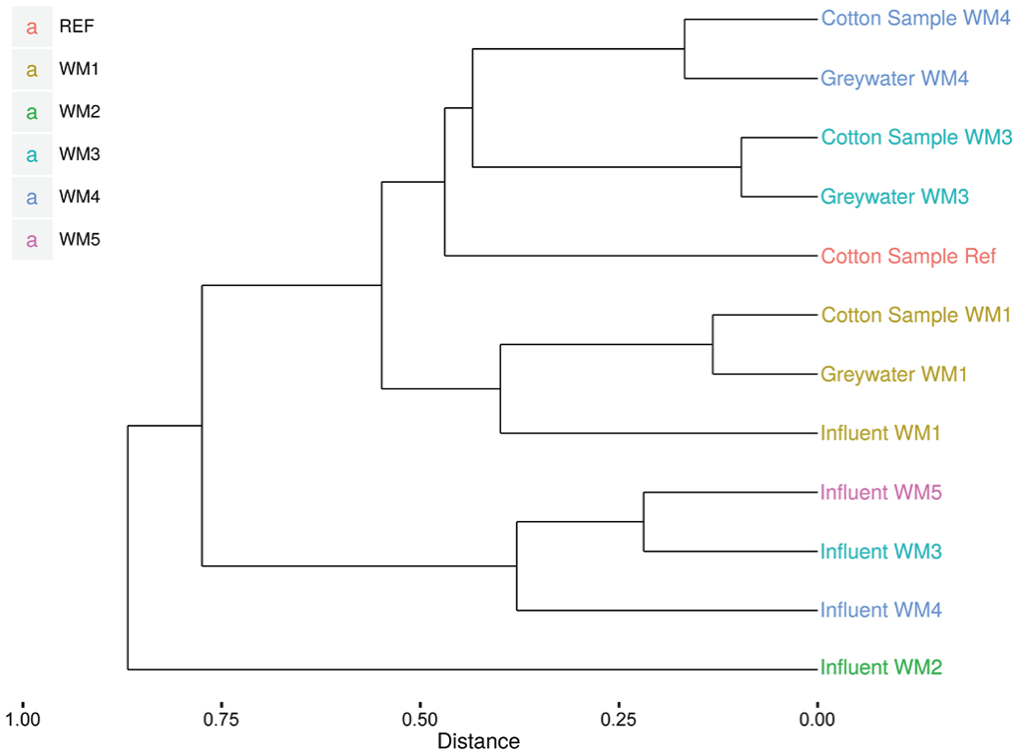
**Clustering of the pyrosequencing results of the different sample types from different household WMs.**Clustering according to the Sorenson index and unweighted pair group with mathematical averages dendrogram method.

**FIGURE 4:**
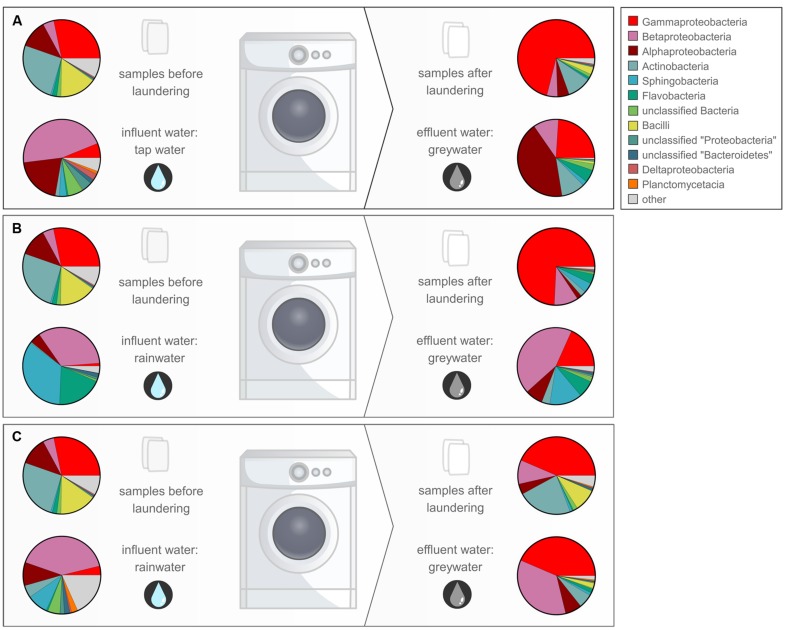
**Overview of the relative abundant bacterial classes in the different samples for WM1 (A), WM3 (B), and WM4 (C) displayed according to the study design**.

In- and outgoing samples from different WMs were collected and amplified with conserved 16S rRNA gene primers generating ∼515-bp amplicons. Two WMs had tap water as influent water; three other WMs used rainwater (**Supplementary Table S1**). The samples clustered together based on sample type (influent water versus greywater and cotton samples). The greywater and cotton samples clustered together according to the WM (**Figure 3**). Large differences were observed for the different WM influent samples, depending on location. In the tap water samples, 12 unique phyla were observed for both locations. The rainwater samples contained 9–13 unique phyla per sample. A high diversity was observed for the influent samples, with a Shannon diversity index of 5.61 ± 0.45 and Chao1 richness of 1412 ± 576 OTUs (see **Table 1** and **Supplementary Figures S1** and **S2**). The diversity in the rainwater samples was lower, as compared to the tap water samples. Most sequences were assigned to *Proteobacteria* and *Bacteroidetes*, with an average of 59 and 22% of the total microbiome, respectively. *Limnohabitans* sp. were often encountered as relatively abundant species in the rainwater samples (12, 9.5, and 5.3% of the total microbiome for WM4, WM5, WM3, respectively). Also, *Flavobacterium* and *Flectobacillus* sp. were found in relative large quantities in the rainwater sample of WM3. In contrast, lower abundances were noticed in tap water samples, with one exception of *Ferribacterium* sp., which represented 16% of the total tap water microbiome of WM1 (**Figure 4**, **Supplementary Table S2**).

**Table 1:** Detailed α-diversity indices based on 454 pyrosequencing and DGGE results.

		454 pyrosequencing				DGGE	
			
Sample type	Sample ID	*N* (total sequence reads)	Observed richness (sequence reads)	Chao1 richness (sequence reads)	Shannon diversity	Richness (# bands)	Gini evenness
Influent	WM1	3464	453	696	5.28	20	0.67
Influent	WM2	3052	1590	2378	6.37	8	0.82
Influent	WM3	3672	592	1009	5.10	26	0.54
Influent	WM4	4144	802	1629	5.83	33	0.47
Influent	WM5	4260	693	1349	5.45	27	0.58
Cotton sample	Ref	2705	439	696	5.20	n.a.	n.a.
Cotton sample	WM1	4387	561	962	4.50	9	0.82
Cotton sample	WM2	n.a.	n.a.	n.a.	n.a.	11	0.77
Cotton sample	WM3	3089	493	904	3.99	18	0.73
Cotton sample	WM4	6151	693	1250	5.42	15	0.78
Cotton sample	WM5	n.a.	n.a.	n.a.	n.a.	14	0.72
Greywater	WM1	2568	804	1460	5.49	18	0.61
Greywater	WM2	n.a.	n.a.	n.a.	n.a.	22	0.52
Greywater	WM3	4565	768	1754	5.34	24	0.56
Greywater	WM4	2726	632	1299	4.92	25	0.62
Greywater	WM5	n.a.	n.a.	n.a.	n.a.	16	0.68


*n.a., not available.*

The washed and unwashed cotton samples contained 8 to 12 unique phyla per sample. The cotton samples overall contained a lower richness of bacteria, with a Chao1 of 953 ± 198 OTUs per sample. The unwashed reference cotton sample contained a comparable richness as the cotton samples washed in the WMs. The reference cotton sample contained a microbiome probably due to the handling. The cotton samples had a Shannon diversity index of 4.78 ± 0.57. Species with high relative abundances were found in the cotton samples with an average of 2 OTUs representing ∼30% of the total bacterial abundance, ranging from 29% for the unwashed to 72% for washed WM3 cotton sample. The majority of OTUs belonged to the *Proteobacteria* (77%), *Actinobacteria* (12%), and *Firmicutes* phylum (5.4%). Within the *Proteobacteria* phylum, *Enhydrobacter*, and *Acinetobacter* were the genera with the highest abundances (up to 67 and 24% of the total microbiome, respectively). Also the Gram-positive *Propionibacterium*, *Staphylococcus*, *Corynebacterium*, and *Micrococcus* bacteria were present in relative abundant quantities (**Table 2**). Remarkably, the same dominant phyla occurred in the unwashed reference cotton sample (*Proteobacteria*, 47%; *Actinobacteria*, 26%, and *Firmicutes*, 22%). The unwashed cotton sample contained a similar diversity as in the laundered cotton samples. Other frequently identified species included *Pseudomonas* sp., *Flavobacterium* sp., *Sphingomonas* sp., *Albidiferax* sp., *Brevundimonas* sp., *Limnohabitans* sp., *Janthinobacterium* sp. and *Flectobacillus* sp., although in lower quantities.

**Table 2:** Relative abundances of the abundant genera from assembled contigs in the cotton samples.

Phylum	Genus		Unwashed cotton sample	Cotton sample WM1	Cotton sample WM3	Cotton sample WM4
*Proteobacteria*	*Enhydrobacter*	G–	2.07%	47.45%	66.66%	5.31%
*Proteobacteria*	*Acinetobacter*	G–	19.45%	15.29%	6.31%	24.34%
*Actinobacteria*	*Propionibacterium*	G+	9.50%	0.71%	0.09%	6.22%
*Firmicutes*	*Staphylococcus*	G+	4.29%	2.62%	0.04%	7.74%
*Actinobacteria*	*Corynebacterium*	G+	3.36%	1.98%	0.04%	8.00%
*Actinobacteria*	*Micrococcus*	G+	2.48%	2.30%	1.34%	0.84%
*Proteobacteria*	*Pseudomonas*	G–	1.96%	0.93%	0.04%	2.65%
*Bacteroidetes*	*Flavobacterium*	G–	0.30%	0.64%	3.20%	0.10%
*Proteobacteria*	*Sphingomonas*	G–	3.55%	0.00%	0.04%	0.23%
*Proteobacteria*	*Albidiferax*	G–	0.00%	0.00%	2.41%	0.62%
*Proteobacteria*	*Brevundimonas*	G–	2.03%	0.32%	0.00%	0.13%
*Proteobacteria*	*Limnohabitans*	G–	0.00%	0.00%	0.39%	1.94%
*Proteobacteria*	*Janthinobacterium*	G–	1.29%	0.00%	0.11%	0.52%
*Bacteroidetes*	*Flectobacillus*	G–	0.00%	0.00%	1.29%	0.00%


In the greywater samples, most sequences were assigned to *Proteobacteria* (59%), *Bacteriodetes* (12%), and the *Actinobacteria* (8.7%). The samples contained 8–10 unique phyla per sample. A high diversity was observed for the greywater samples, with a Shannon diversity index of 5.25 ± 0.24 and a Chao1 estimated richness of 1504 ± 188 OTUs per sample. An average of four OTUs represented >20% of the total bacterial abundance. Typically, a mixture of influent water-related bacterial species and skin-related bacterial species was found in the greywater samples. Among the typical skin-related bacteria, *Enhydrobacter*, *Staphylococcus*, and *Corynebacterium* sp. were found in relatively high abundances. Among the typical water-related bacteria, *Aldibiferax*, *Luteolibacter*, *TM7*, *Schlesneria*, *Polynucleobacter*, *Phenylobacterium*, *Flectobacillus*, *Brevundimonas*, and *Flavobacterium* sp. were found in relatively high quantities (**Figure 4**, **Supplementary Tables S2**–**S4**). Also *Pseudomonas* sp. were frequently found in high amounts and were enriched in two of the three WMs eﬄuents, compared to the influent water. *Enhydrobacter* sp. were generally enriched in the greywater, however, this probably originated from the other clothes brought into the WM. Full overview of the species with the highest relative abundances and rarefaction curves can be found in the **Supplementary Figures S3** and **S4**. Detailed α-diversity characteristics are displayed in **Table 1**.

### Bacterial Community Analysis According to the DGGE

**FIGURE 5:**
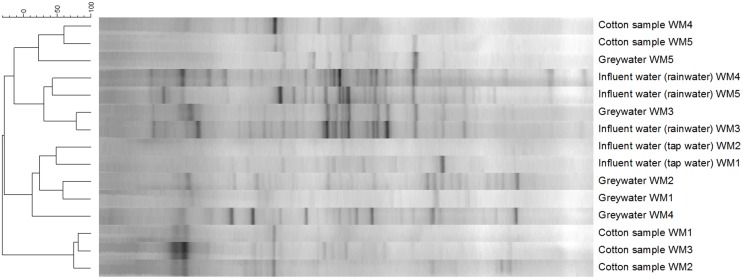
**Clustering of the DGGE bacterial fingerprinting results according to the Pearson correlation and unweighted pair group with mathematical averages dendrogram method**.

The diversity of the samples was characterized by means of the richness, determined as the number of bands observed on gel, and the evenness, calculated as Gini coefficient. Finger printing analyses indicated a large bacterial diversity, mainly in the influent and greywater samples (**Figure 5**). The samples mainly clustered together based on sample type (influent water, greywater, and cotton samples). Relatively low Pearson similarities were found between the different samples from the same household (20 ± 16%). Rainwater influents displayed the highest richness (29 ± 3 bands) and highest evenness (Gini = 0.53 ± 0.04). High Pearson similarities were found between the rainwater influents on the different locations (41 ± 2%). The richness and evenness of tap water samples varied, i.e., 8 and 20 bands with Gini coefficient of 0.82 and 0.67, respectively. Irrespective of the influent water, the eﬄuent greywater displayed comparable richness and evenness (22 ± 4 for tap water and 20 ± 2 bands for rainwater with a Gini coefficient of 0.62 ± 0.05 and 0.57 ± 0.05, respectively). Similar bacterial bands were found in the greywater as in the influent water, indicating a bacterial transfer from influent to greywater (especially for WM3). The cotton samples were determined with the lowest richness (13 ± 3 bands) and the lowest evenness (Gini = 0.76 ± 0.04). The cotton samples clustered separately from the influent and eﬄuent samples, with a high Pearson similarity (44 ± 28%) found within the cotton samples. Although the cotton samples were not used before, certain bacterial bands were associated with the cotton, which were generally not seen in the water samples. Full α-diversity characteristics are displayed in **Table 1**.

## Discussion

Little information exists about the microbial communities in clothes and WMs. In this study, we analyzed the microbial flows in different WMs. Of the 13 phyla detected in the greywater samples, OTUs belonging to the *Proteobacteria* dominated the microbial community. OTUs belonging to the *Bacteroidetes* were another important group in the in- and eﬄuent samples. *Proteobacteria* and *Bacteroidetes* are known for their dominant presence in drinking water supplies (Kwon et al., 2011). For the cotton samples, however, the *Actinobacteria* and the *Firmicutes* were the second and third most important bacterial groups before the *Bacteroidetes*.

The different influent waters used in this study showed major differences. Flow cytometry revealed cell counts of 6–7 × 10^4^ cells ml^-1^ in the tap water, while much higher cell counts were obtained for the rainwater samples, reaching up to 5 × 10^6^ cells ml^-1^. Roof-harvested rainwater can contain a higher concentration of contaminants, due to rinsing of airborne dust, bird faeces, and other organic material, and subsequent collection in the water tank (Kaushik et al., 2014). The identity, diversity, and dominance of the reported genera differed for every household location and water source. The genera present in the rainwater were generally well-known bacteria to inhabit rainwater storage tanks (Evans et al., 2009). The genera detected in the tap water are known to inhabit different environments and were previously reported in aquatic samples, lake sediments, activated sludge, among others (Hong et al., 2010). Opportunistic pathogens were identified in some cases, such as *Leptospira*, *Sphingomonas*, and *Legionella* sp. (Edelstein et al., 2003; Evangelista and Coburn, 2010; Ryan and Adley, 2010), although their abundances were generally not higher than 1% of the total microbiome.

Although low relative bacterial abundances were found in the cotton samples after laundering, a considerable diversity was observed. Certain bacterial bands found on DGGE were associated with cotton, which were generally not observed in the water samples. The initial cotton samples were unused before laundering, but were retrieved with a high diversity of typical skin-, textile- and WM-related bacteria after laundering, as identified by pyrosequencing. In general, all types of bacteria detected on the initial cotton sample were still present in the washed cotton samples (**Figure 4**). Additionally, an apparent enrichment occurred with the skin-related *Enhydrobacter*, *Acinetobacter*, *Corynebacterium*, *Staphylococcus* sp. and the biofilm-related *Pseudomonas* sp. The laundering process resulted in a microbial exchange with other worn clothes and the influent water, with a favored selectivity for typical skin- and clothes-related bacteria. *Micrococcus* sp. were not enriched, however, they were retrieved in abundances of 0.84 to 2.30% in the textile after laundering. Previous research showed that micrococci were generally solely enriched on polyester textiles (Callewaert et al., 2014). Overall, a high occurrence of the *Moraxellaceae* family was apparent in all cotton samples, dominated by *Enhydrobacter* sp. and *Acinetobacter* sp. These were enriched in high amounts in the cotton textiles (up to 67 and 24% of the total textile microbiome, respectively). Both are Gram-negative and ubiquitous skin commensal bacteria (Seifert et al., 1997; Gao et al., 2007). High abundances of *Enhydrobacter* sp. were identified in the greywater, indicating their presence in some people’s skin and clothes microbiome. A recent study showed that *Moraxellaceae* were found among the relative abundant bacteria in the axillary region (Callewaert et al., 2013). *Acinetobacter* sp. are typically retrieved from clothing textiles (Loh et al., 2000; Pilonetto et al., 2004; Callewaert et al., 2014). Only low quantities of *Moraxella* sp. were found in the WMs, which were formerly identified as a malodour causing species in WMs in Japan (Kubota et al., 2012). Additionally, the Gram-positive *Corynebacterium*, *Staphylococcus*, *Propionibacterium*, and *Micrococcus* sp. were identified in relatively high abundances in the laundered cotton samples (up to 8.0, 7.7, 6.2, and 2.3% of the total textile microbiome, respectively). These bacteria are considered as typical skin commensals (Kloos and Musselwhite, 1975; Gao et al., 2007; Grice et al., 2009) and were brought into the WM through the worn clothing textiles. *Corynebacterium* and *Staphylococcus* sp. reside in high quantities in the axillary region (Callewaert et al., 2013), and thus can be transferred in high abundances to the shirts. For WM4, an apparent enrichment of *Corynebacterium* sp. occurred in the laundered cotton piece, suggesting its presence on the skin and textiles of this family. *Corynebacterium* sp. are known to play a key role in body odor development (Leyden et al., 1981). As such, malodorous bacteria can be further distributed to other clothing textiles via the WM. As shown previously, *Staphylococcus* and *Micrococcus* sp. were present in high amounts on clothing textiles (Callewaert et al., 2014). Some of the Gram-positive skin-related species were enriched in the cotton samples, indicating a microbial exchange with other clothing textiles in the WM. None of the typical influent water bacterial species were enriched in the cotton samples. Staphylococci and *Micrococcaceae* were likewise reflected in the greywater samples. For WM4, agar plating indicated 17%, while pyrosequencing indicated 11% staphylococci and *Micrococcaceae* (**Supplementary Table S4**). For WM1 and WM3, agar plating did not indicate any, while pyrosequencing correspondingly found very low numbers of staphylococci and *Micrococcaceae* (1.2 and 0.9%, respectively).

The number of intact bacteria in the greywater was comparable or higher than that of the influent water. The number in greywater ranged from 10^4^ to 10^5^ cells ml^-1^ (plate counts) to 10^6^ cells ml^-1^ (flow cytometry). The TPC were relatively higher for the eﬄuent greywater from WM2 and WM4 (10^5^ CFU ml^-1^ of which 66% were Gram-negative bacteria). The DGGE results of WM2 additionally indicated a large bacterial enrichment from influent to eﬄuent water. This high bacterial concentration might be explained by the fact that WM2 was in use in a household with children and pets in the house. Household dust of houses with indoor pets is shown to carry a more rich and diverse bacterial community (Fujimura et al., 2010), although the effect on absolute cell numbers remains unknown. Among the relative abundant species, no typical dog-related species were found, as compared to literature (Hoffmann et al., 2014). The bacterial community found in the rainwater influent and to a lower extent in the tap water was likewise found in the greywater eﬄuent. A high bacterial transfer was noted from influent to greywater (as seen by the DGGE and pyrosequencing results). Most bacterial species present in the influent water were found in lower quantities in the greywater. As an example, the alpha- and beta-proteobacteria present in the influent water return in high amounts in the eﬄuent water, whilst only marginally present in the cotton samples after laundering (**Figure 4**). It seemed that the water-related bacterial species were generally completely washed out with the greywater and did not adhere to fabrics or WM parts. Nonetheless, certain species were enriched in the greywater, such as *Albidiferax*, *TM7*, *Schlesneria* and *Luteolibacter* sp., which represented more than 10% of the total identified microbiome. Other enriched species included *Sphingomonas*, *Phenylobacterium*, *Brevundimonas*, *Flavobacterium*, *Aquabacterium*, *Polynucleobacter*, *Undibacterium*, and *Legionella* sp. It is suggested that these species formed a biofilm in the WM. All of the enriched bacterial species are indeed known for their biofilm forming capacities (Pang and Liu, 2006; Basson et al., 2008; Miller et al., 2009; Egert et al., 2010; Gattlen et al., 2010; Kwon et al., 2011; Liao et al., 2013; Zhu et al., 2014). Interestingly, the enriched bacterial species differed for every WM, except for *Sphingomonas* sp., which was enriched in two out of three WMs. The pathogenic *Legionella* sp. were enriched in the greywater of one WM (1.25% for WM1), while its presence in the cotton sample was nonetheless very low (0.14%). The latter were not found in the greywater from the other WMs, although they were initially present in low quantities in the influent water. Carry-over of pathogens via WMs was reported before (Wiksell et al., 1973; Munk et al., 2001). *Pseudomonas* sp. were often identified in relative abundant quantities in the cotton samples (up to 2.7% of the total textile microbiome). In the greywater, they were enriched in two of the three WMs, compared to the influent water. It is suggested that these enrichments occurred due to its presence in the WM, as they have been identified as typical biofilm-forming bacteria in WMs (Gattlen et al., 2010). Especially *Pseudomonas putida* sp. were found as important biofilm producers (Gattlen et al., 2010). The biofilm formation can give rise to a number of problems, such as unpleasant odors, fabric staining, and deterioration, reducing the lifetime of the WM or even skin infections (Szostak-Kotowa, 2004). Chemical disinfection (use of bleaching agents), thermal disinfection (use of higher washing temperatures) and/or physical removal (extra centrifuging) can reduce biofilm formation and bacterial loads in the WM. The use of the three washing mechanisms together can have a strong synergistic effect (Arild et al., 2003). In this study, the washing powder did not contain bleach, the laundry machine was operated at low temperatures (30°C) and with a delicate program (no extra centrifuging), which can explain the high (living) bacterial loads retrieved after washing. Although many biofilm-producing bacteria were enriched in the eﬄuent water after laundering, their presence was generally not reflected in the cotton samples. Only a few biofilm forming bacteria, such as *Pseudomonas* and *Sphingomonas* sp., were observed in low quantities in the cotton samples. We can conclude that the water-related biofilms did not transfer their microbial constituents to the laundered clothes. The microbial load brought into the WM by means of the worn clothes seemed more important for the laundered textiles. It seems that certain species have a higher specificity to adhere to clothing textiles.

In this study, four different analysis methods were employed: the molecular-based techniques DGGE and amplicon pyrosequencing, as well as the non-molecular bacterial enumeration techniques agar plating and flow cytometry. DGGE served as a first screening technique for the relative abundant species. Amplicon pyrosequencing added a more thorough insight, with subsequent identification and quantification of the bacterial community (Edwards et al., 2006). Diversity differences between DGGE and amplicon pyrosequencing were observed (**Table 1**). The results from amplicon pyrosequencing were supposed to be more reliable, as DGGE can only visualize bacteria present for at least 1% of the bacterial community (Muyzer and Smalla, 1998). A (weak) positive correlation was observed between the observed richness of the DGGE results with the Chao 1 richness of the pyrosequencing results (see **Supplementary Figurey S5**). The diversity indices derived from DGGE can at least be considered indicative for the diversity found with amplicon pyrosequencing. The molecular-based techniques focused on bacterial DNA from living as well as dead bacterial cells, while the bacterial enumeration techniques focused on the living bacterial cells. When comparing the enumeration techniques, we found large discrepancies between agar plating and flow cytometry. Flow cytometry detected 7 to 157 times more cells in the greywater as compared to plating (**Figure 2**). The agar plating technique differentiated between living, cultivable cells and uncultivable and dead cells. The applied flow cytometry technique differentiated between intact and damaged bacterial cells. It can be concluded that the applied washing program left high amounts of intact bacterial cells in the greywater, whereas only a small portion was able to grow on agar plate. It was previously shown that flow cytometry detects more cells in drinking water samples as compared to plate counts (Hammes et al., 2008), a given also known as “the great plate count anomaly” (Staley and Konopka, 1985). From this study, flow cytometry can be regarded as a useful technique to enumerate bacterial cells.

This study revealed that the household low-temperature laundering process created a bacterial mixing in the laundered clothing textiles. An enrichment of a variety of biofilm-forming bacteria was observed in the studied WMs; however, most of these bacteria were washed out with the greywater. The textiles brought into the WM were found to be more important in the determination of the microbiome of the laundered clothes. The cotton pieces in the WM selected for typical skin- and textile-related bacterial microbiota. Previous research indeed showed that the composition of clothing fibers determined a selective bacterial enrichment (Callewaert et al., 2014). It is suggested that the cause for malodour generation in WMs and clothes is related to the bacteria present in the textiles. It is expected that a household WM plays a role in the specification of the skin microbiome of the household family members. A previous study confirmed that cohabiting family members have large similarities in their –especially skin– microbiome (Song et al., 2013). The laundering process can lead to a mix-up of skin- and clothes-related bacteria between clothes of family members. By means of the WM, malodour-causing microbial species might be further distributed to other clothing textiles. This study gave more insight into the microbial communities and their exchange in household WMs.

## Author Contributions

NB, CC, SVN, F-MK designed the experiments. CC and SVN wrote the main manuscript text. MSG prepared the amplicon libraries and performed the 454 pyrosequencing. F-MK and MSG analyzed the pyrosequencing results. CC and SV performed the experiments and analyzed the flow cytometry, plating and DGGE analyses. All authors reviewed the manuscript.

## Conflict of Interest Statement

The authors declare that the research was conducted in the absence of any commercial or financial relationships that could be construed as a potential conflict of interest.

## Footnotes

1 http://www.r-project.org/

2 http://www.ncbi.nlm.nih.gov/Traces/sra
