# Supplementary material for: Bacterial Exchange in Household Washing Machines
Source: Front Microbiol. 2015 Dec 8;6:1381. doi: 10.3389/fmicb.2015.01381 (PMC4672060; doi:10.3389/fmicb.2015.01381)
Supplement: Supplementary file 1 [file Data_Sheet_1.DOCX]

Supplementary Material

Microbial exchange in household laundry machines

Chris Callewaert^§^, Sam Van Nevel^§^, Frederiek-Maarten Kerckhof, Michael S. Granitsiotis, Nico Boon^*^

^§^ Both authors contributed equally to this work.

*** Correspondence:** Nico Boon, [nico.boon@ugent.be](mailto:nico.boon@ugent.be)

# Supplementary Figures


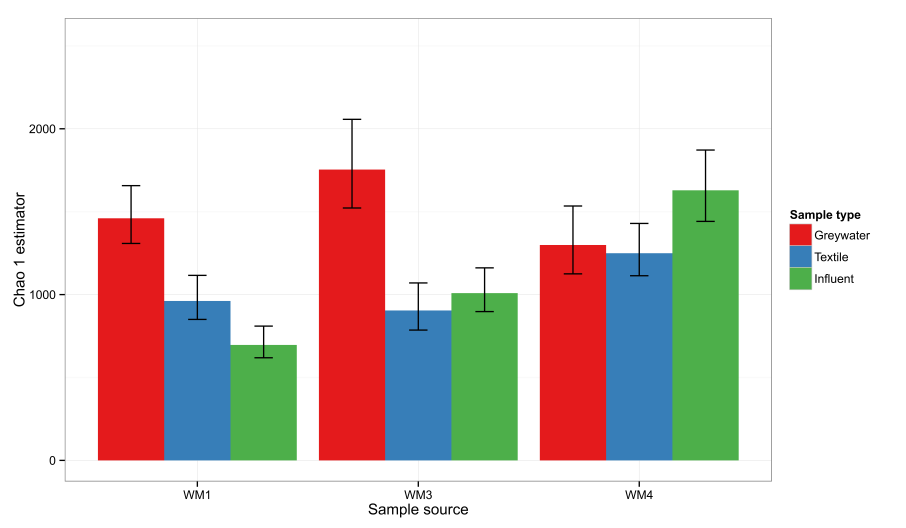


**Figure S1.** Chao1 estimated richness of the samples per laundry machine.


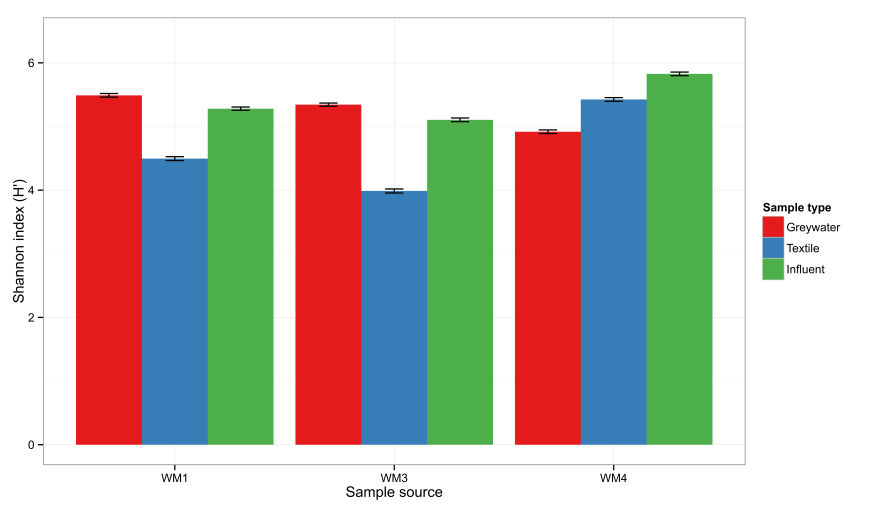


**Figure S2.** Shannon diversity index of the samples per laundry machine.


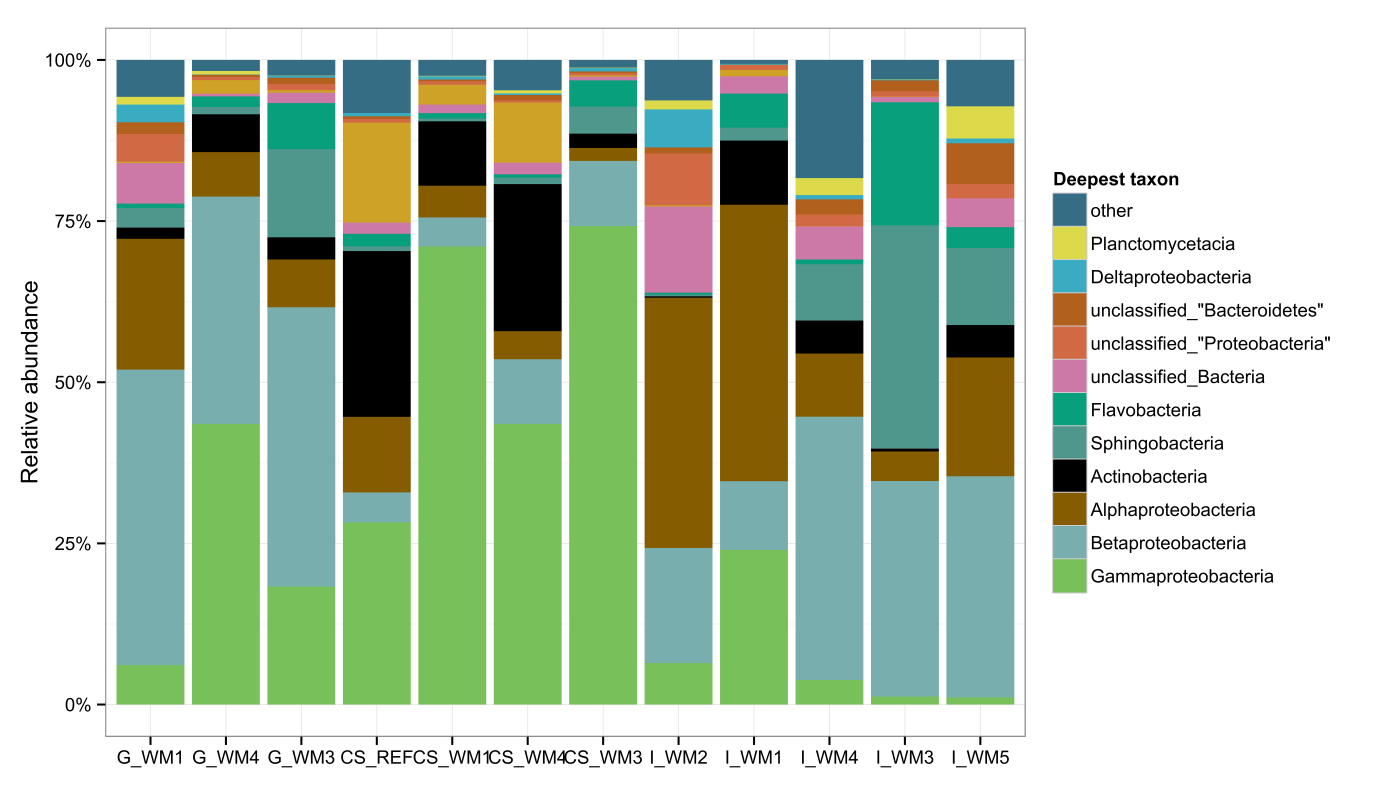


**Figure S3.** Overview of the 12 bacterial classes with the highest abundances in the greywater (G), cotton samples (CS) and influent water (I) samples of the different household washing machines (WM).


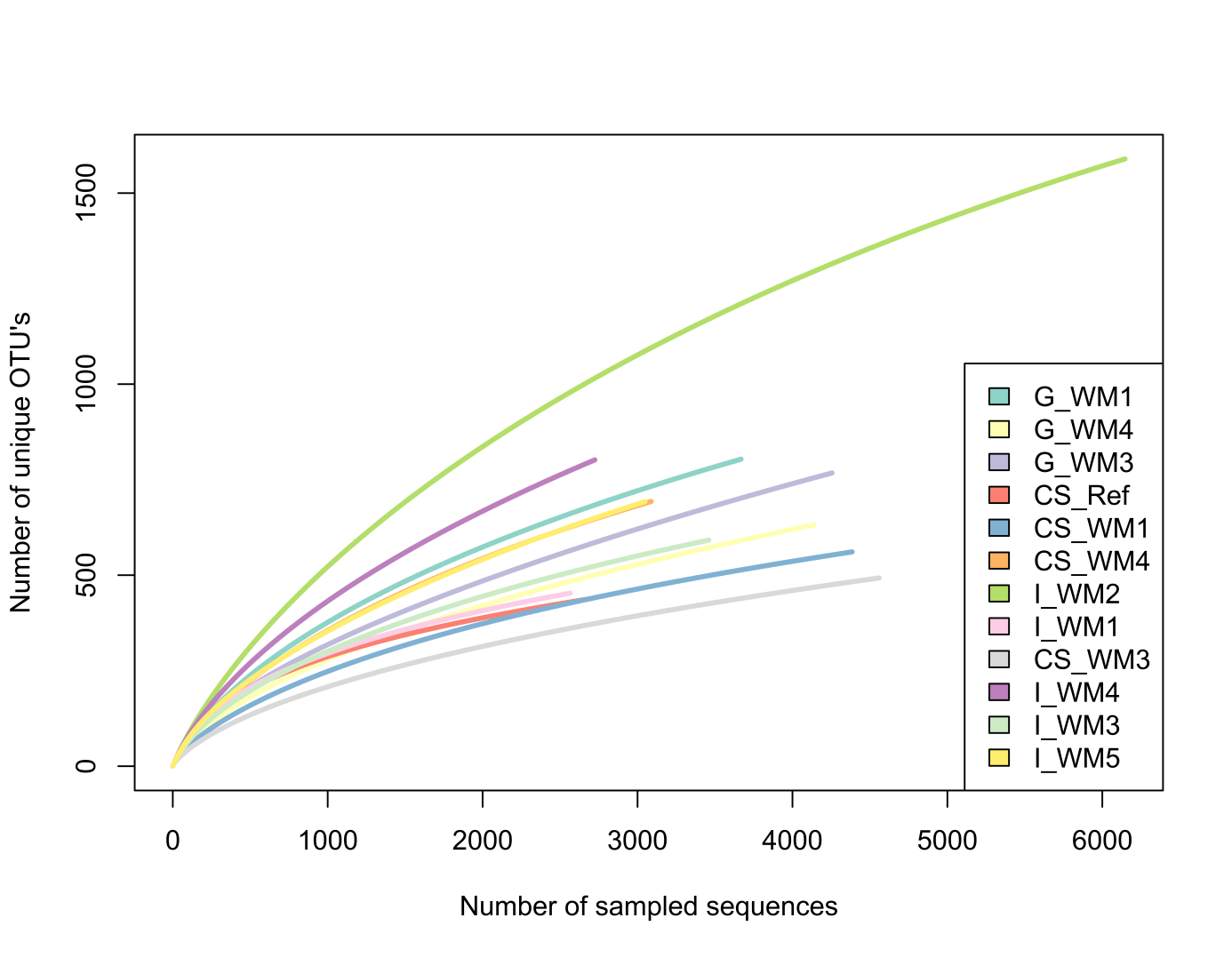


**Figure S4.** Rarefaction curves of the pyrosequenced samples.

**Figure S5.** Relationship between observed richness found for DGGE and Chao 1 richness found for next-generation sequencing (NGS) 454 pyrosequencing.

# Supplementary Tables

**Table S1.** Washing machines (WM) codes and metadata.

| **Code** | **Location** | **Influent water** | **Family** |
| --- | --- | --- | --- |
| WM1 | Gent | Tap water | 1 adult |
| WM2 | Balegem | Tap water | 2 adults, 3 young children, in house pets |
| WM3 | Westrozebeke | Rainwater | 3 adults |
| WM4 | Sijsele | Rainwater | 5 adults, in house pets |
| WM5 | Zulte | Rainwater | 2 adults |

**Table S2.** Relative abundances of the abundant genera from assembled contigs in WM1 (tap water). The upper part of the table is showing the skin/laundry related species while the lower part shows the water related species; - = absent.

|  | **WM1 cotton sample ref** | **WM1 influent water** | **WM1 cotton sample out** | **WM1 greywater** |
| --- | --- | --- | --- | --- |
| *Enhydrobacter* | 2.07% | 0.11% | 47.45% | 12.22% |
| *Acinetobacter* | 19.45% | - | 15.29% | 2.10% |
| *Propionibacterium* | 9.50% | 0.08% | 0.71% | 0.31% |
| *Staphylococcus* | 4.29% | - | 2.62% | 0.97% |
| *Micrococcus* | 2.48% | - | 2.30% | - |
| *Corynebacterium* | 3.36% | - | 1.98% | 0.93% |
| *Pseudomonas* | 1.96% | - | 0.93% | 2.49% |
| *Phenylobacterium* | - | 0.44% | 0.27% | 6.46% |
| *Brevundimonas* | 2.03% | 0.35% | 0.32% | 5.22% |
| *Flavobacterium* | 0.30% | - | 0.64% | 3.46% |
| *Aquabacterium* | - | 1.66% | - | 2.18% |
| *Denitratisoma* | - | 2.62% | 0.62% | - |
| *Ferribacterium* | - | 15.72% | - | - |
| *Afipia* | - | 2.53% | - | 0.39% |
| *Fibrisoma* | - | 2.59% | - | - |
| *Leptospira* | - | 2.21% | - | - |
| *Sphingomonas* | 3.55% | 2.81% | - | 0.43% |

**Table S3.** Relative abundances of the abundant genera from assembled contigs in WM3 (rainwater). The upper part of the table is showing the skin/laundry related species while the lower part shows the water related species; - = absent.

|  | **WM3 cotton sample ref** | **WM3**  **influent water** | **WM3 cotton sample out** | **WM3 greywater** |
| --- | --- | --- | --- | --- |
| *Enhydrobacter* | 2.07% | - | 66.66% | 13.82% |
| *Acinetobacter* | 19.45% | - | 6.31% | 0.12% |
| *Propionibacterium* | 9.50% | - | 0.09% | 0.05% |
| *Staphylococcus* | 4.29% | - | 0.04% | 0.05% |
| *Micrococcus* | 2.48% | - | 1.34% | 0.73% |
| *Corynebacterium* | 3.36% | - | 0.04% | 0.05% |
| *Pseudomonas* | 1.96% | - | 0.04% | 0.19% |
| *Albidiferax* | - | 6.79% | 2.41% | 10.40% |
| *Polynucleobacter* | - | 3.35% | 0.88% | 8.21% |
| *Flavobacterium* | 0.30% | 18.11% | 3.20% | 7.04% |
| *Flectobacillus* | - | 15.39% | 1.29% | 5.52% |
| *Sediminibacterium* | - | 4.10% | 0.39% | 3.03% |
| *Limnohabitans* | - | 5.31% | 0.39% | 2.89% |
| *Janthinobacterium* | 1.29% | 3.75% | 0.11% | 1.36% |
| *Sphingomonas* | 3.55% | 0.35% | 0.04% | 1.43% |

**Table S4.** Relative abundances of the abundant genera from assembled contigs in WM4 (rainwater). The upper part of the table is showing the skin/laundry related species while the lower part shows the water related species; - = absent.

|  | **WM4 cotton sample ref** | **WM4 influent water** | **WM4 cotton sample out** | **WM4 greywater** |
| --- | --- | --- | --- | --- |
| *Acinetobacter* | 19.45% | - | 24.34% | 1.25% |
| *Corynebacterium* | 3.36% | - | 8.00% | 5.62% |
| *Staphylococcus* | 4.29% | - | 7.74% | 9.72% |
| *Propionibacterium* | 9.50% | - | 6.22% | 0.36% |
| *Enhydrobacter* | 2.07% | - | 5.31% | 1.30% |
| *Micrococcus* | 2.48% | - | 0.84% | 0.29% |
| *Pseudomonas* | 1.96% | - | 2.65% | 2.03% |
| *TM7* | - | 0.15% | - | 12.57% |
| *Schlesneria* | - | 2.31% | 0.32% | 12.40% |
| *Luteolibacter* | - | 0.11% | - | 10.23% |
| *Polynucleobacter* | - | 5.61% | 0.74% | 0.92% |
| *Armatimonas_gp1* | - | 3.63% | 0.19% | 0.36% |
| *Nitrospira* | - | 6.12% | 0.19% | 0.10% |
| *Limnohabitans* | - | 12.06% | 1.94% | 0.29% |
| *Sediminibacterium* | - | 2.90% | - | 0.53% |
| *Albidiferax* | - | 2.35% | 0.62% | 0.19% |
| *Undibacterium* | - | 1.83% | 0.16% | 1.83% |
| *Sphingomonas* | 3.55% | 0.37% | 0.23% | 2.56% |
